# Promising results of HIV prevention trials highlight the benefits of collaboration in global health: The perspective of the Forum HIV Recency Assay Working Group

**DOI:** 10.1371/journal.pgph.0003878

**Published:** 2024-10-24

**Authors:** Robin Schaefer, Logan Donaldson, Mitchell Leus, Chukwunomso E. Osakwe, Benjamin Chimukangara, Shona Dalal, Ann Duerr, Fei Gao, David V. Glidden, Beatriz Grinsztejn, Jessica Justman, Grace Kumwenda, Oliver Laeyendecker, Ha Youn Lee, Frank Maldarelli, Kenneth H. Mayer, Jeffrey Murray, Bharat S. Parekh, Brian Rice, Michael N. Robertson, Suzue Saito, Vani Vannappagari, Mitchell Warren, Diana Zeballos, Jörg Zinserling, Veronica Miller

**Affiliations:** 1 Forum for Collaborative Research, University of California, Berkeley, Washington, District of Columbia, United States of America; 2 National Institutes of Health, Bethesda, Maryland, United States of America; 3 Global HIV, Hepatitis, and STIs Programmes, World Health Organization, Geneva, Switzerland; 4 Fred Hutchinson Cancer Center, Seattle, Washington, United States of America; 5 University of California, San Francisco, California, United States of America; 6 Instituto Nacional de Infectologia Evandro Chagas, Fundação Oswaldo Cruz (INI-Fiocruz), Rio de Janeiro, Brazil; 7 ICAP at Columbia University, Mailman School of Public Health, New York, New York, United States of America; 8 AVAC, Southern Region, Malawi; 9 National Institute of Allergy and Infectious Diseases, National Institutes of Health, Bethesda, Maryland, United States of America; 10 Keck School of Medicine, University of Southern California, Los Angeles, California, United States of America; 11 National Cancer Institute, Bethesda, Maryland, United States of America; 12 The Fenway Institute, Fenway Health, Boston, Massachusetts, United States of America; 13 Department of Medicine, Beth Israel Deaconess Medical Center/Harvard Medical School, Boston, Massachusetts, United States of America; 14 Independent consultant, Lewes, Delaware, United States of America; 15 Division of Global HIV & TB, Centers for Disease Control and Prevention, Atlanta, Georgia, United States of America; 16 The University of Sheffield, Sheffield, United Kingdom; 17 BDI Consulting, Nipomo, California, United States of America; 18 ViiV Healthcare, Durham, North Carolina, United States of America; 19 AVAC, New York, New York, United States of America; 20 Instituto de Saúde Coletiva, Universidade Federal da Bahia, Salvador, Bahia, Brazil; 21 Bundesinstitut für Arzneimittel und Medizinprodukte, Bonn, Germany; PLOS: Public Library of Science, UNITED STATES OF AMERICA

Twelve years ago, in 2012, the US Food and Drug Administration (FDA) approved oral tenofovir disoproxil fumarate (TDF) and emtricitabine (FTC) as pre-exposure prophylaxis (PrEP) for HIV prevention. In 2015, the World Health Organization (WHO) recommended offering oral PrEP to all people at substantial HIV risk. In 2023, there were 3.5 million PrEP users globally [1], up from 600,000 users in 2019 [2]. However, progress varies significantly across regions, countries, and populations, and further increases are needed to meet the 21.2 million PrEP user target by the end of 2025 [1]. Only Eastern and Southern Africa is on track to meet its target of 3.6 million users (2.4 million in 2023). Furthermore, in 2023, 1.3 million people acquired HIV globally [1], underscoring the ongoing need to improve access to prevention options and develop new products that align with user needs and preferences. In this article, we argue that collaboration between diverse stakeholders is necessary to address critical issues in the HIV response and advance global health (Box 1).

**Box 1. Key messages**

- **Clinical trial challenges:** The increasing number of efficacious HIV prevention products challenges the feasibility of conducting HIV prevention clinical trials, requiring adjustments in how trials are conducted.
- **Consensus-building:** The Forum for Collaborative Research facilitated a consensus among diverse stakeholders–including regulatory agencies, academia, industry, and community–on HIV prevention trial design, proposing to use background HIV incidence, estimated with the HIV recency assay, as a comparator.
- **Implementation of a novel design:** This trial design was implemented in large phase 3 clinical trials on six-monthly injections with lenacapavir for HIV prevention, which found this product highly efficacious and safe.
- **Collaboration matters:** These trials underscore the importance of collaboration in global health. By providing an independent space and emphasizing a common goal (enabling future HIV prevention trials), a consensus could be reached among stakeholders who may have conflicting views.

**Improving global health:** Unlocking this ‘collaborative advantage’ is needed to end HIV epidemics globally and improve global public health.

Other PrEP products have become available. In 2019, the US FDA approved oral tenofovir alafenamide (TAF) and FTC for some populations. In 2021, WHO recommended the dapivirine in a vaginal ring (DVR) for women. The US approved cabotegravir as the first long-acting injectable PrEP (CAB-LA) in 2021 (recommended by WHO in 2022). In June 2024, Gilead Sciences announced that an independent data monitoring committee (DMC) recommended discontinuing the blinded phase of PURPOSE 1, a phase 3 HIV prevention clinical trial among cisgender women in South Africa and Uganda investigating six-monthly injections with lenacapavir, a capsid inhibitor, and offering open-label lenacapavir to all participants [3]. In September, the DMC of the PURPOSE 2 trial of lenacapavir among cisgender men and trans and gender-diverse people who have sex with men in seven countries also recommended offering lenacapavir to all participants [4]. These recommendations were based on interim analyses finding lenacapavir to be safe and highly efficacious at preventing HIV acquisition. Further clinical trials of lenacapavir are ongoing, and various other products are in development [5].

The US FDA approval of TDF/FTC, and the WHO DVR recommendation, were based on placebo-controlled randomized trials, the gold standard to establish safety and efficacy of biomedical products. However, the evolving HIV prevention landscape necessitated adjustments in how clinical trials are conducted. Placebo-controlled trials for PrEP are no longer ethical [6], as prevention standards include several evidence-based options–oral PrEP, the DVR, injectable PrEP–depending on clinical indications and local availability. Therefore, clinical trials need to compare investigational agents against control groups with participants being offered active biomedical prevention options. Such active-control study designs were adopted in the non-inferiority [7] and superiority [8] clinical trials forming the basis for CAB-LA regulatory approvals. However, with increasingly efficacious standards of prevention, active-control trials face significant challenges, including difficulties in establishing non-inferiority margins and large sample size requirements [9,10]. This challenges the feasibility of HIV prevention clinical trials.

The Forum for Collaborative Research (Forum), at the University of California, Berkeley, creates, maintains, and directs networks of diverse stakeholders to address pressing issues in global health (https://forumresearch.org/). Building on decades of experience in fostering consensus around drug development and clinical trials, the Forum established a working group on HIV prevention trial design challenges and designs of future trials. The working group, including representatives from regulatory agencies, multilateral organizations, academia, industry, and communities affected by HIV, published a consensus statement on these deliberations [10]. The group recommended utilizing background HIV incidence as a comparator for HIV incidence observed in the trial group receiving the investigational product. The HIV recency assay was proposed as one method to estimate background HIV incidence in the absence of PrEP. This approach involves estimating HIV incidence cross-sectionally using a recent infection testing algorithm among individuals screened for study participation and found living with HIV. The well-characterized and validated limiting antigen avidity assay (LAg) is the recency assay most widely used [11]. Newer approaches, such as high-throughput genomic incidence assays, could further increase the accuracy of incidence estimations [12], although these have not been tested in surveillance or clinical trial settings. The consensus statement outlined challenges with the proposed approach. This includes ensuring that individuals contributing to the background HIV incidence estimate and those enrolled in the trial are well-matched in terms of HIV acquisition risk, and that the willingness of individuals to be tested for HIV during the screening process is independent of HIV status.

This study design was adopted in the clinical trials investigating injectable lenacapavir for PrEP (PURPOSE 1 and 2). It was also adopted in the IMPOWER trials investigating once-monthly oral islatravir for PrEP before the program was stopped [13]. The primary comparator in the PURPOSE trials was the background HIV incidence estimated with the recency assay. PURPOSE 1 found zero HIV acquisitions among 2,134 individuals who received lenacapavir, compared to a background HIV incidence of 2.41 per 100 person-years [3]. In PURPOSE 2, two of 2,180 participants receiving lenacapavir acquired HIV (incidence of 0.10 per 100 person-years), compared to a background incidence of 2.37 per 100 person-years [4]. Both trials found a statistical superiority to the HIV incidence among those randomized to oral TDF/FTC (1.69 and 0.93 per 100 person-years in PURPOSE 1 and 2, respectively).

PURPOSE 1 and 2 are the first HIV prevention trials demonstrating the feasibility of using background HIV incidence for primary comparison. The success of the PURPOSE trials in establishing efficacy of an investigational product highlights the benefits of, and need for, collaboration in global health. Drug development is often a competitive process. The Forum enabled an alternative model by providing a neutral platform for discussion in which all relevant stakeholders were included with an equal voice. This independent space, in which disagreements could openly be aired, built trust between stakeholders who may have conflicting views. The emphasis on a common goal to create a public benefit–enabling HIV prevention clinical trials and improving public health–further built trust and ensured that value created by the working group was distributed among all stakeholders, not just benefitting specific parties.

This collaborative process facilitated consensus on a novel trial design. Now, PURPOSE 1 and 2 demonstrated high efficacy of a new PrEP product that can become an important additional HIV prevention tool. When approved by regulatory agencies and widely available, lenacapavir expands the armamentarium of HIV prevention options for people who could benefit from PrEP. More options are needed so that users can choose what suits their needs and preferences, improving PrEP uptake and effective use, to halt new HIV infections that remain unacceptably high among many populations [14].

Various HIV prevention products are in pre-clinical and clinical development, including additional PrEP options, broadly neutralizing antibodies, and vaccines [5]. As more potentially efficacious products progress in development and become available, clinical trial designs to establish comparative efficacy will become more challenging. Therefore, we must learn from the successes and challenges of the PURPOSE trials and similar studies and build consensus around innovative trial designs, which may involve background HIV incidence estimates, data from active-control trial groups, or a combination of the two [15]. The Forum continues to provide a platform to deliberate HIV prevention trial designs and remains committed to its mission to unlock the ‘collaborative advantage’ that benefits all stakeholders and is needed to end HIV epidemics globally.
