# Correction: Promising results of HIV prevention trials highlight the benefits of collaboration in global health: The perspective of the Forum HIV Recency Assay Working Group

**DOI:** 10.1371/journal.pgph.0007622

**Published:** 2026-11-13

**Authors:** The PLOS Global Public Health Staff

## Notice of Republication

This article was republished on January 24, 2025, to correct formatting errors in the bullet points in Box 1. The publisher apologizes for the errors. Please download this article again to view the correct version. The originally published, uncorrected article and the republished, corrected articles are provided here for reference.

## Supporting information

S1 File Originally published, uncorrected article. (PDF)

S2 File Republished, corrected article. (PDF)
