# Supplementary material for: Correction: Promising results of HIV prevention trials highlight the benefits of collaboration in global health: The perspective of the Forum HIV Recency Assay Working Group
Source: PLOS Glob Public Health. 2026 Nov 13;6(11):e0007622. doi: 10.1371/journal.pgph.0007622 (PMC13766251; doi:10.1371/journal.pgph.0007622)
Supplement: Supplementary file 2 [file pgph.0007622.s002.pdf]

OPINION

Promising results of HIV prevention trials highlight the benefits of collaboration in global health: The perspective of the Forum HIV Recency Assay Working Group

Robin Schaefer[1]*, Logan Donaldson[1], Mitchell Leus[1], Chukwunomso E. Osakwe[1], Benjamin Chimukangara[2], Shona Dalal[3], Ann Duerr[4], Fei Gao[4], David V. Glidden[5], Beatriz Grinsztejn[6], Jessica Justman[7], Grace Kumwenda[8], Oliver Laeyendecker[9], Ha Youn Lee[10], Frank Maldarelli[11], Kenneth H. Mayer[12,13], Jeffrey Murray[14], Bharat S. Parekh[15], Brian Rice[16], Michael N. Robertson[17], Suzue Saito[7], Vani Vannappagari[18], Mitchell Warren[19], Diana Zeballos[20], Jörg Zinserling[21], Veronica Miller[1]

1 Forum for Collaborative Research, University of California, Berkeley, Washington, District of Columbia, United States of America, **2** National Institutes of Health, Bethesda, Maryland, United States of America, **3** Global HIV, Hepatitis, and STIs Programmes, World Health Organization, Geneva, Switzerland, **4** Fred Hutchinson Cancer Center, Seattle, Washington, United States of America, **5** University of California, San Francisco, California, United States of America, **6** Instituto Nacional de Infectologia Evandro Chagas, Fundação Oswaldo Cruz (INI-Fiocruz), Rio de Janeiro, Brazil, **7** ICAP at Columbia University, Mailman School of Public Health, New York, New York, United States of America, **8** AVAC, Southern Region, Malawi, **9** National Institute of Allergy and Infectious Diseases, National Institutes of Health, Bethesda, Maryland, United States of America, **10** Keck School of Medicine, University of Southern California, Los Angeles, California, United States of America, **11** National Cancer Institute, Bethesda, Maryland, United States of America, **12** The Fenway Institute, Fenway Health, Boston, Massachusetts, United States of America, **13** Department of Medicine, Beth Israel Deaconess Medical Center/Harvard Medical School, Boston, Massachusetts, United States of America, **14** Independent consultant, Lewes, Delaware, United States of America, **15** Division of Global HIV & TB, Centers for Disease Control and Prevention, Atlanta, Georgia, United States of America, **16** The University of Sheffield, Sheffield, United Kingdom, **17** BDI Consulting, Nipomo, California, United States of America, **18** ViiV Healthcare, Durham, North Carolina, United States of America, **19** AVAC, New York, New York, United States of America, **20** Instituto de Saúde Coletiva, Universidade Federal da Bahia, Salvador, Bahia, Brazil, **21** Bundesinstitut für Arzneimittel und Medizinprodukte, Bonn, Germany

* rschaefer@forumresearch.org

Check for updates

OPEN ACCESS

Citation: Schaefer R, Donaldson L, Leus M, Osakwe CE, Chimukangara B, Dalal S, et al. (2024) Promising results of HIV prevention trials highlight the benefits of collaboration in global health: The perspective of the Forum HIV Recency Assay Working Group. PLOS Glob Public Health 4(10): e0003878. https://doi.org/10.1371/journal.pgph.0003878

Editor: Julia Robinson, PLOS: Public Library of Science, UNITED STATES OF AMERICA

Published: October 24, 2024

Copyright: This is an open access article, free of all copyright, and may be freely reproduced, distributed, transmitted, modified, built upon, or otherwise used by anyone for any lawful purpose. The work is made available under the Creative Commons CC0 public domain dedication.

Funding: This work was supported, in whole or in part, by the Bill & Melinda Gates Foundation [INV-045445]. Under the grant conditions of the Foundation, a Creative Commons Attribution 4.0 Generic License has already been assigned to the Author Accepted Manuscript version that might arise from this submission. The Forum for Collaborative Research, as an organization, also receives grants from the pharmaceutical industry but these are unrestricted and not specific to the work described in this article. Additional support was provided by the Division of Intramural Research, National Institute of Allergy and

Twelve years ago, in 2012, the US Food and Drug Administration (FDA) approved oral tenofovir disoproxil fumarate (TDF) and emtricitabine (FTC) as pre-exposure prophylaxis (PrEP) for HIV prevention. In 2015, the World Health Organization (WHO) recommended offering oral PrEP to all people at substantial HIV risk. In 2023, there were 3.5 million PrEP users globally [1], up from 600,000 users in 2019 [2]. However, progress varies significantly across regions, countries, and populations, and further increases are needed to meet the 21.2 million PrEP user target by the end of 2025 [1]. Only Eastern and Southern Africa is on track to meet its target of 3.6 million users (2.4 million in 2023). Furthermore, in 2023, 1.3 million people acquired HIV globally [1], underscoring the ongoing need to improve access to prevention options and develop new products that align with user needs and preferences. In this article, we argue that collaboration between diverse stakeholders is necessary to address critical issues in the HIV response and advance global health (Box 1).

Infectious Diseases, National Institutes of Health. The sponsors of this work contributed to the discussions in the working group meetings that led to this article's development; however, the sponsors had no role in writing the article and deciding to submit the paper for publication. The authors alone are responsible for the views expressed in this article and they do not necessarily represent the views, decisions, or policies of the institutions with which they are affiliated.

Competing interests: We have read the journal's policy and the authors of this manuscript have the following competing interests to declare: Forum for Collaborative Research, as an organization, receives grants from the pharmaceutical industry but these are unrestricted and not specific to the work described in this article. KHM has received unrestricted research grants from Gilead Sciences, Merck & Co., Inc., and ViiV Healthcare. JM is a paid consultant for both Gilead Sciences in the area of HIV prevention. BSP, as an inventor of the LAg-Avidity EIA at CDC, receives a portion of the royalties from the sale of the test kits, as per policy of the U.S. government. MNR received consulting fees from the Bill and Melinda Gates Foundation, Merck, the Forum for Collaborative research, and the International AIDS Vaccine Initiative in 2024. VV is a full-time employee of ViiV Healthcare and owns GSK stock. HYL received NIH NIAID grant support for genomic HIV incidence assay research. All other authors have declared that no competing interests exist.

Box 1. Key messages

- **Clinical trial challenges:** The increasing number of efficacious HIV prevention products challenges the feasibility of conducting HIV prevention clinical trials, requiring adjustments in how trials are conducted.
- **Consensus-building:** The Forum for Collaborative Research facilitated a consensus among diverse stakeholders–including regulatory agencies, academia, industry, and community–on HIV prevention trial design, proposing to use background HIV incidence, estimated with the HIV recency assay, as a comparator.
- **Implementation of a novel design:** This trial design was implemented in large phase 3 clinical trials on six-monthly injections with lenacapavir for HIV prevention, which found this product highly efficacious and safe.
- **Collaboration matters:** These trials underscore the importance of collaboration in global health. By providing an independent space and emphasizing a common goal (enabling future HIV prevention trials), a consensus could be reached among stakeholders who may have conflicting views.
- **Improving global health:** Unlocking this 'collaborative advantage' is needed to end HIV epidemics globally and improve global public health.

Other PrEP products have become available. In 2019, the US FDA approved oral tenofovir alafenamide (TAF) and FTC for some populations. In 2021, WHO recommended the dapivirine in a vaginal ring (DVR) for women. The US approved cabotegravir as the first long-acting injectable PrEP (CAB-LA) in 2021 (recommended by WHO in 2022). In June 2024, Gilead Sciences announced that an independent data monitoring committee (DMC) recommended discontinuing the blinded phase of PURPOSE 1, a phase 3 HIV prevention clinical trial among cisgender women in South Africa and Uganda investigating six-monthly injections with lenacapavir, a capsid inhibitor, and offering open-label lenacapavir to all participants [3]. In September, the DMC of the PURPOSE 2 trial of lenacapavir among cisgender men and trans and gender-diverse people who have sex with men in seven countries also recommended offering lenacapavir to all participants [4]. These recommendations were based on interim analyses finding lenacapavir to be safe and highly efficacious at preventing HIV acquisition. Further clinical trials of lenacapavir are ongoing, and various other products are in development [5].

The US FDA approval of TDF/FTC, and the WHO DVR recommendation, were based on placebo-controlled randomized trials, the gold standard to establish safety and efficacy of biomedical products. However, the evolving HIV prevention landscape necessitated adjustments in how clinical trials are conducted. Placebo-controlled trials for PrEP are no longer ethical [6], as prevention standards include several evidence-based options–oral PrEP, the DVR, injectable PrEP–depending on clinical indications and local availability. Therefore, clinical trials need to compare investigational agents against control groups with participants being offered active biomedical prevention options. Such active-control study designs were adopted in the non-inferiority [7] and superiority [8] clinical trials forming the basis for CAB-LA regulatory approvals. However, with increasingly efficacious standards of prevention, active-control trials face significant challenges, including difficulties in establishing non-inferiority margins and large sample size requirements [9,10]. This challenges the feasibility of HIV prevention clinical trials.

The Forum for Collaborative Research (Forum), at the University of California, Berkeley, creates, maintains, and directs networks of diverse stakeholders to address pressing issues in global health (https://forumresearch.org/). Building on decades of experience in fostering consensus around drug development and clinical trials, the Forum established a working group on HIV prevention trial design challenges and designs of future trials. The working group, including representatives from regulatory agencies, multilateral organizations, academia, industry, and communities affected by HIV, published a consensus statement on these deliberations [10]. The group recommended utilizing background HIV incidence as a comparator for HIV incidence observed in the trial group receiving the investigational product. The HIV recency assay was proposed as one method to estimate background HIV incidence in the absence of PrEP. This approach involves estimating HIV incidence cross-sectionally using a recent infection testing algorithm among individuals screened for study participation and found living with HIV. The well-characterized and validated limiting antigen avidity assay (LAg) is the recency assay most widely used [11]. Newer approaches, such as high-throughput genomic incidence assays, could further increase the accuracy of incidence estimations [12], although these have not been tested in surveillance or clinical trial settings. The consensus statement outlined challenges with the proposed approach. This includes ensuring that individuals contributing to the background HIV incidence estimate and those enrolled in the trial are well-matched in terms of HIV acquisition risk, and that the willingness of individuals to be tested for HIV during the screening process is independent of HIV status.

This study design was adopted in the clinical trials investigating injectable lenacapavir for PrEP (PURPOSE 1 and 2). It was also adopted in the IMPOWER trials investigating once-monthly oral islatravir for PrEP before the program was stopped [13]. The primary comparator in the PURPOSE trials was the background HIV incidence estimated with the recency assay. PURPOSE 1 found zero HIV acquisitions among 2,134 individuals who received lenacapavir, compared to a background HIV incidence of 2.41 per 100 person-years [3]. In PURPOSE 2, two of 2,180 participants receiving lenacapavir acquired HIV (incidence of 0.10 per 100 person-years), compared to a background incidence of 2.37 per 100 person-years [4]. Both trials found a statistical superiority to the HIV incidence among those randomized to oral TDF/FTC (1.69 and 0.93 per 100 person-years in PURPOSE 1 and 2, respectively).

PURPOSE 1 and 2 are the first HIV prevention trials demonstrating the feasibility of using background HIV incidence for primary comparison. The success of the PURPOSE trials in establishing efficacy of an investigational product highlights the benefits of, and need for, collaboration in global health. Drug development is often a competitive process. The Forum enabled an alternative model by providing a neutral platform for discussion in which all relevant stakeholders were included with an equal voice. This independent space, in which disagreements could openly be aired, built trust between stakeholders who may have conflicting views. The emphasis on a common goal to create a public benefit–enabling HIV prevention clinical trials and improving public health–further built trust and ensured that value created by the working group was distributed among all stakeholders, not just benefitting specific parties.

This collaborative process facilitated consensus on a novel trial design. Now, PURPOSE 1 and 2 demonstrated high efficacy of a new PrEP product that can become an important additional HIV prevention tool. When approved by regulatory agencies and widely available, lenacapavir expands the armamentarium of HIV prevention options for people who could benefit from PrEP. More options are needed so that users can choose what suits their needs and preferences, improving PrEP uptake and effective use, to halt new HIV infections that remain unacceptably high among many populations [14].

Various HIV prevention products are in pre-clinical and clinical development, including additional PrEP options, broadly neutralizing antibodies, and vaccines [5]. As more potentially efficacious products progress in development and become available, clinical trial designs to establish comparative efficacy will become more challenging. Therefore, we must learn from the successes and challenges of the PURPOSE trials and similar studies and build consensus around innovative trial designs, which may involve background HIV incidence estimates, data from active-control trial groups, or a combination of the two [15]. The Forum continues to provide a platform to deliberate HIV prevention trial designs and remains committed to its mission to unlock the 'collaborative advantage' that benefits all stakeholders and is needed to end HIV epidemics globally.

Acknowledgments

Between 2021 and 2022, the Forum for Collaborative Research convened the Prevention Trials in Women Working Group and the HIV Recency Assay Working Group, which contributed to the content discussed in this article. In 2024, the HIV Recency Assay Working Group was re-established and contributed to the development of this article. We thank all working group members, past and present, for their contributions.

Author Contributions

Conceptualization: Robin Schaefer, Logan Donaldson, Mitchell Leus, Chukwunomso E. Osakwe, Benjamin Chimukangara, Shona Dalal, Ann Duerr, Fei Gao, David V. Glidden, Beatriz Grinsztejn, Jessica Justman, Grace Kumwenda, Oliver Laeyendecker, Ha Youn Lee, Frank Maldarelli, Kenneth H. Mayer, Jeffrey Murray, Bharat S. Parekh, Brian Rice, Michael N. Robertson, Suzue Saito, Vani Vannappagari, Mitchell Warren, Diana Zeballos, Jörg Zinserling, Veronica Miller.

Funding acquisition: Veronica Miller.

Project administration: Robin Schaefer, Logan Donaldson, Mitchell Leus, Chukwunomso E. Osakwe, Veronica Miller.

Supervision: Robin Schaefer, Veronica Miller.

Writing – original draft: Robin Schaefer.

Writing – review & editing: Robin Schaefer, Logan Donaldson, Mitchell Leus, Chukwunomso E. Osakwe, Benjamin Chimukangara, Shona Dalal, Ann Duerr, Fei Gao, David V. Glidden, Beatriz Grinsztejn, Jessica Justman, Grace Kumwenda, Oliver Laeyendecker, Ha Youn Lee, Frank Maldarelli, Kenneth H. Mayer, Jeffrey Murray, Bharat S. Parekh, Brian Rice, Michael N. Robertson, Suzue Saito, Vani Vannappagari, Mitchell Warren, Diana Zeballos, Jörg Zinserling, Veronica Miller.

References

1. Joint United Nations Programme on HIV/AIDS (UNAIDS), The urgency of now: AIDS at a crossroads. 2024 Global AIDS Update. Geneva: UNAIDS; 2024.
2. Schaefer R, Schmidt H-MA, Ravasi G, Mozalevskis A, Rewari BB, Lule F, et al. Adoption of guidelines on and use of oral pre-exposure prophylaxis: a global summary and forecasting study. The Lancet HIV. 2021; 8(8):e502–e510. https://doi.org/10.1016/S2352-3018(21)00127-2 PMID: 34265283
3. Bekker L-G, Das M, Abdool Karim Q, Ahmed K, Batting J, Brumskine W, et al. Twice-Yearly Lenacapavir or Daily F/TAF for HIV Prevention in Cisgender Women. New England Journal of Medicine. 2024; 391:1179–1192. https://doi.org/10.1056/NEJMoa2407001 PMID: 39046157

4. Gilead Sciences. Gilead's Twice-Yearly Lenacapavir for HIV Prevention Reduced HIV Infections by 96% and Demonstrated Superiority to Daily Truvada® in Second Pivotal Phase 3 Trial. 2024 September 12, 2024 [Cited 2024 September 26]. Available from: https://www.gilead.com/news/news-details/2024/gileads-twiceyearly-lenacapavir-for-hiv-prevention-reduced-hiv-infections-by-96-and-demonstrated-superiority-to-daily-truvada.

5. Beyrer C, Tomaras GD, Gelderblom HC, Gray GE, Janes HE, Bekker L-G, et al. Is HIV epidemic control by 2030 realistic? The Lancet HIV. 2024; 11(7):e489–e494. https://doi.org/10.1016/S2352-3018(24)00098-5 PMID: 38925732

6. Brown BJ, Sugarman J. Why ethics guidance needs to be updated for contemporary HIV prevention research. Journal of the International AIDS Society. 2020; 23(5):e25500. https://doi.org/10.1002/jia2.25500 PMID: 32406990

7. Landovitz Raphael J, Donnell D, Clement Meredith E, Hanscom B, Cottle L, Coelho L, et al. Cabotegravir for HIV Prevention in Cisgender Men and Transgender Women. New England Journal of Medicine. 2021; 385(7):595–608. https://doi.org/10.1056/NEJMoa2101016 PMID: 34379922

8. Delany-Moretlwe S, Hughes JP, Bock P, Ouma SG, Hunidzarira P, Kalonji D, et al. Cabotegravir for the prevention of HIV-1 in women: results from HPTN 084, a phase 3, randomised clinical trial. The Lancet. 2022; 399(10337):1779–1789. https://doi.org/10.1016/S0140-6736(22)00538-4 PMID: 35378077

9. Cutrell A, Donnell D, Dunn DT, Glidden DV, Grobler A, Hanscom B, et al. HIV prevention trial design in an era of effective pre-exposure prophylaxis. HIV Clin Trials. 2017; 18(5–6):177–188. https://doi.org/10.1080/15284336.2017.1379676 PMID: 29039265

10. Parkin N, Gao F, Grebe E, Cutrell A, Das M, Donnell D, et al. Facilitating Next-Generation Pre-Exposure Prophylaxis Clinical Trials Using HIV Recent Infection Assays: A Consensus Statement from the Forum HIV Prevention Trial Design Project. Clinical Pharmacology & Therapeutics. 2023; 114(1):29–40. https://doi.org/10.1002/cpt.2830 PMID: 36550769

11. Kin-On Lau J, Murdock N, Murray J, Justman J, Parkin N, Miller V. A systematic review of limiting antigen avidity enzyme immunoassay for detection of recent HIV-1 infection to expand supported applications. Journal of Virus Eradication. 2022; 8(3):100085. https://doi.org/10.1016/j.jve.2022.100085 PMID: 36124229

12. Faraci G, Park SY, Love TMT, Dubé MP, Lee HY. Precision detection of recent HIV infections using high-throughput genomic incidence assay. Microbiol Spectr. 2023; 11(5):e0228523. https://doi.org/10.1128/spectrum.02285-23 PMID: 37712639

13. Merck & Co., Inc. Merck to Initiate New Phase 3 Clinical Program with Lower Dose of Daily Oral Islatravir in Combination with Doravirine for Treatment of People with HIV-1 Infection. 2022 September 20, 2022 [Cited 2024 July 2]. Available from: https://www.merck.com/news/merck-to-initiate-new-phase-3-clinical-program-with-lower-dose-of-daily-oral-islatravir-in-combination-with-doravirine-for-treatment-of-people-with-hiv-1-infection/.

14. Bekker L-G, Pike C, Hillier SL. HIV prevention: better choice for better coverage. Journal of the International AIDS Society. 2022; 25(1):e25872. https://doi.org/10.1002/jia2.25872 PMID: 35030296

15. Gao F, Janes HE, Buchbinder S, Donnell D. Active-Controlled Trial Design for HIV Prevention Trials with a Counterfactual Placebo. arXiv:2407.04812 [Preprint]. 2024 [cited 2024 July 2]. Available from: https://www.arxiv.org/abs/2407.04812.
